# Disseminated gonococcal infection in an immunocompetent young man

**DOI:** 10.1590/0037-8682-0368-2022

**Published:** 2022-12-16

**Authors:** Gabriel Castro Tavares, Cecília Schubert Xavier Lagalhard Victer

**Affiliations:** 1 Universidade Federal do Rio de Janeiro, Hospital Universitário Clementino Fraga Filho, Serviço de Dermatologia, Rio de Janeiro, RJ, Brasil.

**FIGURE 1:**
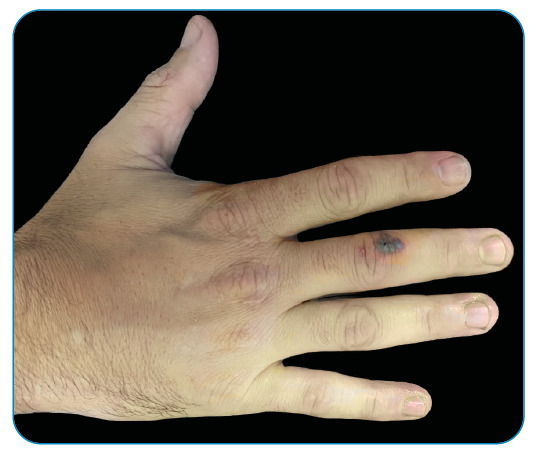
Vesicles with hemorrhagic content at the dorsum of the right middle finger.

A 35-year-old man from Rio de Janeiro, reported painful skin lesions breaking out on his right hand, seven days prior, in addition to fever and pain while walking. He presented with no history of trauma, headache, dysuria, urethral discharge, any comorbidities or use of medication, and admitted engaging in unprotected sex in the previous month. On physical examination, he was normotensive, with normal heart rate, and febrile (39°C). Vesicles with hemorrhagic content were observed on an erythematous base in the palmar region and dorsum of the right middle finger (Figure 1). Arthralgia in the right knee and wrists, and tenosynovitis in the left calcaneal tendon were noted. Blood count showed leukocytosis (25,500/mm³) and neutrophilia (20,660/ mm³). Rapid tests for HIV, syphilis, and hepatitis B and C were negative. The clinical diagnosis was disseminated gonococcal infection. The patient was hospitalized, blood cultures were negative, intravenous ceftriaxone 1g/day for seven days, and oral azithromycin 1g in a single dose were administered. The clinical-laboratory status showed complete improvement after three days of antibiotic therapy. Disseminated gonococcal infection is a medical emergency and its treatment should not be dependent on isolation of *Neisseria gonorrhoeae*, as laboratory tests may have low sensitivity. The case reinforces the importance of clinical diagnosis showing the triad, “tenosynovitis, polyarthralgia, and dermatitis"^1^ ^,^ ^2^. Early treatment prevents serious complications such as liver abscess, osteomyelitis, endocarditis, meningitis, and death^3^.
